# Structural Insights into Spare-Tire DNA G‑Quadruplex from the Human *VEGF* Promoter

**DOI:** 10.1021/acschembio.5c00226

**Published:** 2025-05-16

**Authors:** Ines Burkhart, Vivien Rose McKenney, Julia Wirmer-Bartoschek, J. Tassilo Grün, Alexander Heckel, Harald Schwalbe

**Affiliations:** † Institute for Organic Chemistry and Chemical Biology, Center for Biomolecular Magnetic Resonance (BMRZ), Goethe University Frankfurt am Main, Max-von-Laue-Str. 7, Frankfurt/Main, Hesse 60438, Germany; ‡ Institute for Organic Chemistry and Chemical Biology, 9173 Goethe-University Frankfurt, Max-von-Laue-Str. 7, Frankfurt/Main, Hesse 60438, Germany

## Abstract

The vascular endothelial growth factor (*VEGF*) promoter region, which is involved in cancer progression, contains guanine-rich sequences capable of forming G-quadruplex (G4) structures. G4s play a critical role in transcriptional regulation and genomic stability and exhibit high structural polymorphism. The major *VEGF* G4 adopts a parallel topology involving the first four of five G-tracts (*VEGF*1234), while a potential “spare-tire” mechanism suggests the formation of *VEGF*1245 in response to oxidative damage. Here, we characterize this alternative G4 (*VEGF*1245), formed by excluding the third G-tract, using circular dichroism and nuclear magnetic resonance spectroscopy. Structural analysis reveals that *VEGF*1245 folds in a hybrid conformation. Different from the other five tracts containing G4s, for which various strand topologies can rapidly interconvert, *VEGF*1245 remains thermodynamically metastable and does not refold spontaneously into *VEGF*1234 at physiological temperatures. Further trapping of the *VEGF*1245 conformation by a photolabile protecting group and its in situ release documents that the transition to *VEGF*1234 requires elevated temperatures, implicating kinetic barriers in the refolding process and the delineation of *VEGF*1245 as a prominent metastable conformation. Our findings provide new insights into transcriptional regulation and DNA repair for cancer-related *VEGF*-G4.

## Graphical abstract

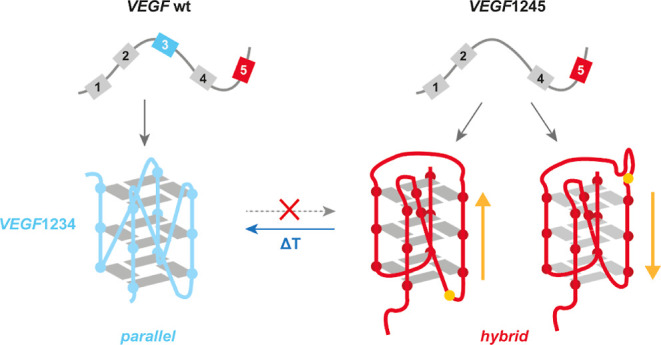

## Introduction

Angiogenesis, the process of new blood vessel formation, is a critical factor in cancer progression and is primarily regulated by the vascular endothelial growth factor (*VEGF*).^1^ The promoter region of *VEGF* contains guanine-rich sequences capable of forming DNA G-quadruplexes (G4s).^2^ G4s are secondary structures composed of stacked guanine tetrads stabilized by Hoogsteen hydrogen bonding and monovalent cations such as K^+^ or Na^+^.^3^ They have been identified not only in oncogene promoters but also in other key genomic regions, including telomeres and replication origins.^4,5^ Their structural polymorphism,^6^ influenced by sequence and cation type, leads to complex folding landscapes.

The major thermodynamically favored *VEGF* promoter G4, which plays a crucial role in transcriptional regulation, consists of four consecutive G-tracts. Specific constructs of this G-rich sequence have been shown to adopt a parallel topology.^7^ This structure originates from a region spanning nucleotides −85 to −50 relative to the transcription initiation site, a region critical for *VEGF* promoter activity.^8^ The wild-type *VEGF* sequence contains, however, five G-tracts, with the fourth and fifth tracks separated by a seven-nucleotide loop containing two deoxyguanosines. While the fifth G-tract is not involved in the most stable G4 fold, studies by Fleming et al. suggested that it may function as a “spare tire”, replacing a damaged G-tract to maintain G4 integrity.^9^ This putative replacement is particularly relevant in the context of oxidative stress, as guanine-rich sequences are highly susceptible to oxidative damage from reactive oxygen species (ROS).^10,11^ The efficient repair of such damage is dependent on base excision repair pathways involving glycosylases. However, within the G4 core, these repair mechanisms are attenuated, which underscores the functional importance of the fifth G-tract in facilitating access to glycosylases and ensuring DNA integrity. This effect was also demonstrated for the human nei-like DNA glycosylase 3 (NEIL3) promoter region.^12^ Additionally, the oxidation of *VEGF* was found to affect recognition by nucleolin and influenced the G4 topology.^13^ In the context of a duplex-G4-duplex motif, the major *VEGF* G4 appears to exhibit accelerated folding kinetics in response to DNA damage.^14^ Given the significance of G4 structures in genomic stability and transcriptional regulation, elucidating their role in DNA repair and damage recognition has direct implications for cancer therapeutics. In some cases, it is imperative to identify specific G4 topologies for effective DNA repair mechanisms.^15^ While in the *c-MYC* promoter, alternative G4 isomers^16,17^ have been extensively studied using time-resolved NMR spectroscopy to elucidate their folding pathway,^18,19^ further investigations are required including the current study of *VEGF* G4s to underline the generality of the spare-tire mechanism for G4 folding despite oxidative damage.

Currently, the structure of the thermodynamically most stable *VEGF* G4 has been reported.^7^ This study focused on nonwild-type sequence optimized for NMR structural studies. A comparison of the CD spectra of the wild-type *VEGF* sequence and the previously studied major *VEGF* G4 reveals a residual peak at 290 nm, suggesting the presence of additional G4 polymorphs, including antiparallel or hybrid topologies beyond the predominant parallel G4.

We thus studied in detail the wild-type *VEGF* G4 to demonstrate that the G4 of *VEGF* involving tracks 1, 2, 4, and 5 forms a hybrid quadruplex. This *VEGF*1245 is a metastable polymorph with remarkable thermostability; it is capable of refolding into *VEGF*1234 only after being heated to elevated temperatures (*T* = 50 °C). Furthermore, investigation of sequences with successively introduced deoxyguanosines in the third G-tract further stresses the complexity of the folding landscape in *VEGF* quadruplexes, as already introduction of a single G in the third track shifts the conformation from hybrid to a multitude of different conformations. A thorough understanding of G4 folding dynamics is therefore essential not only for understanding the molecular mechanism of G4s protection to inhibit cancer progression but also for advancing therapeutic strategies in cancer treatment.

## Results and Discussion

Distinct G4 structures identified in promoter regions have been investigated by X-ray crystallography^20^ and NMR spectroscopy.^7,17,21–28^ When only one conformation constituted of three tetrads is present, signals of 12 imino hydrogens are visible in the Hoogsteen fingerprint area of a 1D ^1^H NMR spectrum. This NMR signature documents that a given G4 folds into a single, distinct topology: parallel, antiparallel, or hybrid. When a sequence is able to form more than one distinct G4, more than 12 imino signals can occur in the NMR spectrum. The occurrence of an additional set of NMR resonances demonstrates the coexistence of several slowly interconverting G4 conformations. From quantification of unequal signal intensities, the relative stabilities of the different conformations at equilibrium can be derived in principle. In practice, the chemical shift resolution is often not sufficient, and we thus apply here a combinatorial labeling strategy, developed previously for rapid RNA NMR resonance assignment.^29^

The *VEGF* wild-type sequence is a highly polymorphic structure with five G-tracts and at least 7 possible G-register shift isomers (Figure 1). To explore possible tract isomers of *VEGF*, we first tested oligonucleotides that differ in G-tracts and loop lengths compared to the wild-type (wt) sequence (Figure 1, Table 1). The major *VEGF* G4 folds from the first four G-tracts (*VEGF*1234 short) with a parallel topology and three loops. *VEGF* harbors five G-tracts that can be involved in quadruplex folding, thus we examined *VEGF* without either the first G-tract (*VEGF*2345) or the third G-tract (*VEGF*1245). In addition to spare-tire isomerism, G-register shifts can also occur as a source of polymorphism in quadruplexes. G-register shifts can appear when a G-tract harbors more than 3 deoxyguanosines. As only three deoxyguanosines within one tract can participate in a three tetrad G4, the relevant tract can shift up and down, increasing the number of possible isomers.^30^ As the third tract is in the middle of the sequence, the deoxyguanosines were replaced with thymidines to prevent the formation of Hoogsteen bonds. We also investigated the influence of the long 7 nt loop in *VEGF* wt and how the G4 topology changes when the loop is shortened. Figure 2 shows the CD spectra of the sequences listed in Table 1. The spectrum of wild-type *VEGF* shows a maximum at around 260 nm and a minimum at 240 nm, which is typical for parallel G4. In addition, a local maximum at around 290 nm is observed, typical for antiparallel conformations. Removal of the loop from the wild-type sequence leads to a decrease in the antiparallel fraction in the CD-spectrum (shoulder at 290 nm; Figure 2A). Melting analysis showed that the shortening of the loop in the wt decreased the melting point of the parallel species (Table 2). Based on these analyses, we can deduce that the long loop favors antiparallel topologies as the major conformation and hybrid topologies as the minor conformation. When the long loop is added to *VEGF*1234 short, the stability decreases slightly and the overall structure does not change. Interestingly, when the first G-tract is removed, the CD-signature shows a more pronounced antiparallel contribution, which has the same height as the parallel peak. In this case, the long loop construct is less stable than the short loop construct. However, analysis of the sequences by 1D ^1^H NMR spectroscopy revealed highly polymorphic structures that do not appear to fold into a single distinct G4 fold (Figure S4). The sequence lacking the third G-tract, which has a hybrid CD-signature (Figure 2), showed distinct imino resonances in the 1D ^1^H NMR spectrum.

**Figure 1:**
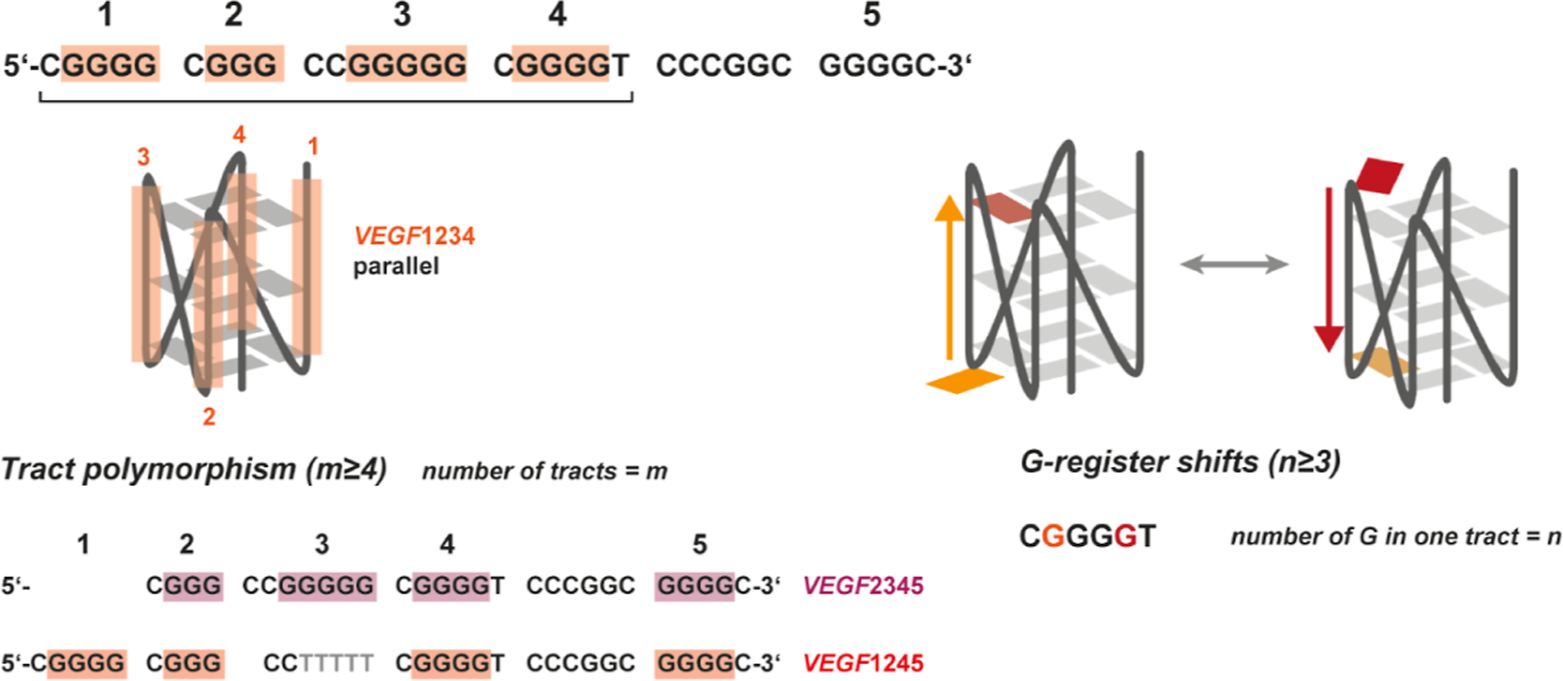
Schematic representation of the investigated G4 from the *VEGF* promoter region. *VEGF* preferably folds into the parallel G4 formed by the G-tracts 1–4. We here explored possible spare-tire isomers which exclude the first or the third G-tract of the G4. The number of deoxyguanosine nucleotides in each of the tracks differs and *n* ≥ 3, giving rise to register shifts. Register shifted tracks differ in the incorporation of which of the more than three deoxyguanosine nucleotides participate in the G tetrad.

**Table 1:** Oligonucleotide Sequences from the Investigated *VEGF* Tract and Loop Isomers

name	sequence					
*VEGF* wild-type (loop)	5′-CGGGG	CGGG	CCGGGGG	CGGGG	TCCCGGCC	GGGGC-3′
*VEGF*12345 short	5′-CGGGG	CGGG	CCGGGGG	CGGGG	T	GGGGC-3′
*VEGF*1234 loop	5′-CGGGG	CGGG	CCGGGGG	CGGGG	TCCCGGCC-3′	
*VEGF*1234 short	5′-CGGGG	CGGG	CCGGGGG	CGGGG	T-3′	
*VEGF*2345 loop		5′-CGGG	CCGGGGG	CGGGG	TCCCGGCC	GGGGC-3′
*VEGF*2345 short		5′-CGGG	CCGGGGG	CGGGG	T	GGGGC-3′
*VEGF*1245	5′-CGGGG	CGGG	CCTTTTT	CGGGG	TCCCGGCC	GGGGC-3′

**Figure 2:**
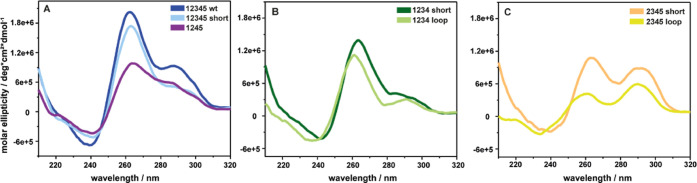
CD spectra of wild-type *VEGF*, *VEGF*12345 short, and *VEGF*1245 (A, blue and purple), the major *VEGF* quadruplex *VEGF*1234 and *VEGF*1234 loop (B, green), and the short-/long-looped variants of *VEGF*2345 (C, yellow).

**Table 2:** Melting Points Determined from CD-Detected Melted Analysis for the Constructs Shown in Figure 1

sequence	melting point/°C
*VEGF* wild-type (loop)	71.4 ± 0.5
*VEGF*12345 short	64.4 ± 0.2
*VEGF*1234 loop	62.8 ± 0.7
*VEGF*1234 short	66.9 ± 0.1
*VEGF*2345 loop	n.d.
*VEGF*2345 short	62.3 ± 0.5
*VEGF*1245	87.4 ± 0.8

**Table 3:** Used Bruker NMR Spectrometers with Respective Probe Heads

spectrometer/MHz	probe head
AVIII 600 HD	Cryoprobe-TCI Prodigy ^1^H [^13^C, ^15^N]
AVIII 700 HD	Cryoprobe-QCI ^1^H [^13^C, ^15^N, ^31^P]
AVIII 800	Cryoprobe-TXO ^13^C, ^15^N [^1^H]
AVIII 800 HD	Cryoprobe-TCI ^1^H [^13^C, ^15^N]

Thus, *VEGF*1245 is able to fold into a stable G4 structure with a melting point of 87.4 °C. To determine the arrangement of the deoxyguanosines in the tetrads, ^15^N- and ^13^C-labeled oligonucleotides^31^ were purchased to implement the combinatorial labeling approach as proposed by Klingler et al.^29^ Chemical synthesis allows targeted introduction of ^13^C, ^15^N-labeled deoxyguanosines in multiple samples, which allowed us to assign all NMR signals unambiguously by applying a procedure that excludes specific peaks as they do not appear in ^13^C, ^15^N-filtered experiments (Figure 3A). ^15^N-filtered 1D ^1^H NMR spectra were obtained for each sample (Figure 3B). The assignment was achieved by the comparison of ^1^H, ^15^N-BEST-TROSY and ^1^H, ^13^C-HSQC spectra (Figures S2 and S3).^32,33^ The 1D ^1^H NMR spectrum shows more than 12 imino resonances, unambiguously showing that *VEGF*1245 adopts several long-lived G4 topologies in slow exchange. Further, no imino signal for ^13^C, ^15^N-labeled G2 could be detected, allowing us to conclude that tract 1 consists of the deoxyguanosines G3–G4–G5. The imino and amino proton chemical shift assignments obtained from the ^13^C, ^15^N-filtered heteronuclear correlation experiments were transferred to the ^1^H, ^1^H-NOESY spectrum to gain information about the tetrad topologies. Between the imino resonances, strong and weak cross-peaks were detected. The analysis of these cross-peaks and their intensities shows that the tracts track 1: G3–G4–G5 and track 2: G7–G8–G9 run antiparallel.

**Figure 3:**
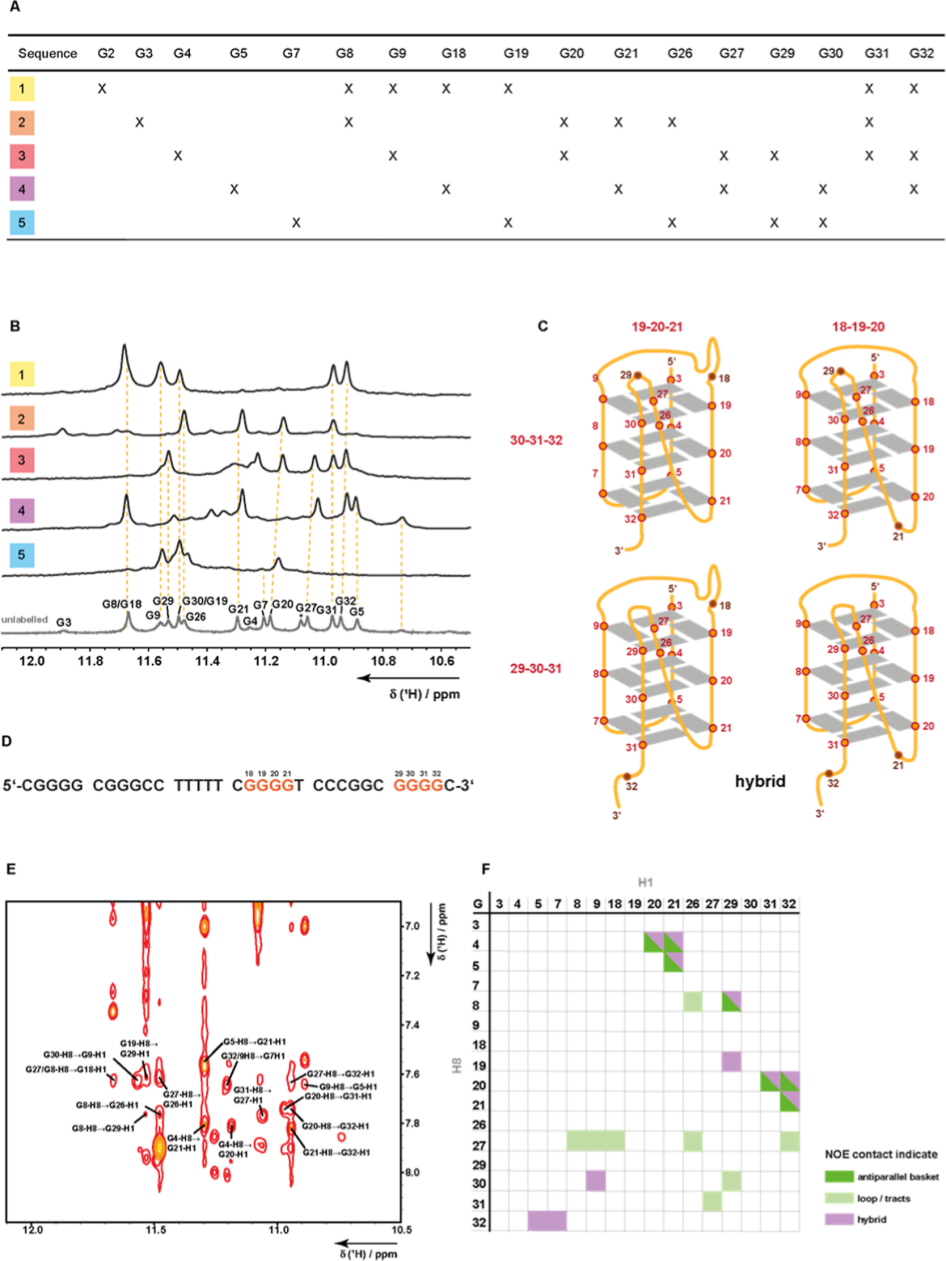
(A) Sequences of five selective *VEGF*1245 samples with their labeling scheme. Samples contain ^13^C- and ^15^N-labeled nucleobases at the positions marked with a X. (B) Assigned 1D ^1^H NMR imino proton region of unlabeled *VEGF*1245 (bottom) and ^15^N-filtered 1D ^1^H NMR spectra of each labeled sample. (C) Schematic representation of the G4s formed by *VEGF*1245, based on the CD and NMR data. The sequences of G-tracts 4 and 5 contain more than three deoxyguanosines, creating the possibility of multiple conformations due to G-register shifts. (D) Sequence of *VEGF*1245 with annotated guanines that are able to form G-register isomers. (E) Imino-aromatic proton correlations from the 2D ^1^H, ^1^H-NOESY experiment. (F) Tabular representation of the found imino-aromatic proton contacts with annotation what structure the respective contact is indicating. The NOE contacts show some contacts that can be assigned to a hybrid or an antiparallel form; however, four contacts can only be assigned to a hybrid fold.

Imino–imino and imino-aromatic contacts were analyzed to obtain information about the tetrad arrangement. G5, G7, G21, and G32 form a tetrad as they have imino–imino and aromatic-imino contacts to each other. Interestingly, an additional NOE contact between G32 and G19 is observed, pointing to a possible G-register shift isomer. The observation that for all four deoxyguanosines of tracts 4 and 5 an imino signal can be detected further proves the occurrence of G-register dynamics for *VEGF*1245 (Figure 3D). Furthermore, G18 has contacts with G4 and G9, suggesting participation in the upper tetrad. The middle tetrad consists of G4, G8, G31, and G20, as evidenced by NOE contacts of G20-H8 → G31-H1 and G4-H8 → G20-H1. Furthermore, the nucleotides of this tetrad also have NOE contacts with deoxyguanosines of the upper and lower tetrads (G20 → G30, G31 → G9, G8 → G29 (G-register when tract V is shifted), and G4 → G18 (G-register when tract 4 is shifted)). The lower tetrad consists of G3, G9, G19, and G30. It is notable that the imino resonances of G19 and G30 exhibit a significant overlap. Additionally, G19 has contacts with G29, suggesting a G-register shift of the fifth tract. This tetrad appears to be the most dynamic, as no imino–imino contacts can be detected between the participating imino resonances, but numerous contacts were observed with the middle tetrad. Within consecutive G-tracts, NOE contacts were detected to show that G7, G8, and G9 are sequentially connected. Furthermore, G26 and G27 also have a detectable imino peak, although they were not located in any of the G-tracts. We interpret these results to be consistent with the formation of a long loop structure that is stacked onto the structure. When considering the aromatic-imino region (Figure 3E), G27 and G26 are located in close proximity to G32, G31, G8, and G9. This observation indicates the potential for loop stacking on the side of the G-quadruplex, which is formed by tracts 2 and 5. The sequential walk in the aromatic-sugar region of the NOESY was partly possible. Within the G-tracts, cross-peaks were detected, while no connectivity was detected between the tracts, suggesting a higher flexibility of the loops. Taking into account all NOEs, G19–G20–G21 and G30–G31–G32 appear to form G-tracts 4 and 5. As we found four aromatic-imino NOE contacts, which can only be explained with a hybrid topology (Figure 3F), we propose the respective schematic representation of the *VEGF*1245 G4 structure in Figure 3C including all possible G-register isomers. In order to ascertain the stability of the identified hybrid G-quadruplex, deoxyguanosines were successively reintroduced into the third G-tract, and the resulting sequences were investigated by 1D ^1^H NMR and CD spectroscopy (Figure 4). The imino resonance pattern was only maintained by complete deletion of the third G-tract. Even with reintroduction of a single guanosine, the CD pattern indicates the presence of a hybrid/parallel mixture, while the 1D ^1^H NMR spectrum mirrors that of the *VEGF* wild-type sequence. Notably, the signals between 11.4 and 11.6 ppm undergo a substantial shift in the presence of one or more deoxyguanosines in the third tract. These signals belong to G9, G29, G30, G19, and G26, which are all located at the upper tetrad. These signals can be used as a marker for *VEGF*1245 (marked in red, Figure 4), as the other imino resonances exhibit an excessively large overlay, thereby hindering clear distinction.

**Figure 4:**
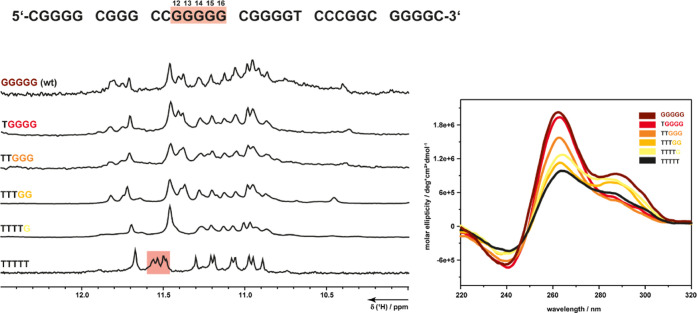
1D ^1^H NMR and CD spectra of *VEGF*1245 (black) and *VEGF*1245 variants, successively introducing one G into the third G-tract (yellow to brown). Both methods show that only with complete deletion of the third G-tract, the unique imino pattern belonging to the hybrid G4 is formed. After addition of one G into the positions of the third G-tract, the sequence shows the same pattern like the wild-type sequence of *VEGF*. Introduction of more G’s in the third G-tract also elevates the parallel proportion in the CD spectrum.

This finding suggests that the thermodynamically favored state of *VEGF*1234 is readily accessible by the addition of at least one guanosine in the third G-tract. It remains unclear how fast this transition occurs and whether folding intermediates are involved in the refolding process.

To investigate the kinetic behavior of this transition and its impact on the folding landscape of *VEGF* G4s, a *VEGF*1245 construct was synthesized in which the involvement of the third G-tract is not excluded by incorporation of thymidines but by photolabile protecting groups (PPGs). In strong contrast to the invasive G to T mutation, PPGs block interactions present in the wild-type sequence, but the wild-type sequence can be restored upon light irradiation. The attachment of PPGs to nucleobases hinders the formation of hydrogen bonds and, consequently, Hoogsteen base pairing. For photocaging, a diethylaminocoumarin (DEACM) photocage^34^ was introduced to protect deoxyguanosine at the O6 position and to trap the *VEGF*1245 quadruplex. After irradiation at λ = 355 nm, the third G-tract is restored and the sequence is identical to the wild-type *VEGF* sequence (Figure 5A).

The DEACM-caged oligonucleotide with a blocked third G-tract has a similar imino proton pattern as *VEGF*1245 and adopts the same topology. It is important to note that the spectral pattern of the DEACM-caged oligonucleotide is slightly different compared to *VEGF*1245. We believe that this is due to the slight alteration in the chemical environment consequent to the incorporation of DEACM. The presence of 10 distinct imino resonances indicates the formation of a single major G4 topology, which is formed by tracts 1, 2, 4, and 5. After irradiation, the G4 pattern remained unchanged, suggesting that G4 did not refold to *VEGF*1234. At temperatures of 25 and 37 °C, only a slight broadening of the resonances was observed, suggesting that the folding ensemble becomes more dynamic. Refolding to the more stable *VEGF*1234 could not be observed even after several days at 37 °C. However, heating the sample for several minutes at 50 °C, which resulted in a broadened imino region, led to the observation of signal shifts to the *VEGF*1234 (illustrated in blue in Figure 5A,B). This finding indicates that a refolding process is necessary, which involves the breakage and subsequent reconstitution of the hydrogen bonds. It is important to note that this process becomes possible only at elevated temperatures, when molecular motion is increased and the DNA is more dynamic. Subsequent slow cooling to RT resulted in the retention of the uncaged DNA in the *VEGF*1234 quadruplex fold. Therefore, folding from *VEGF*1245 to *VEGF*1234 is slow and kinetically inhibited.

**Figure 5:**
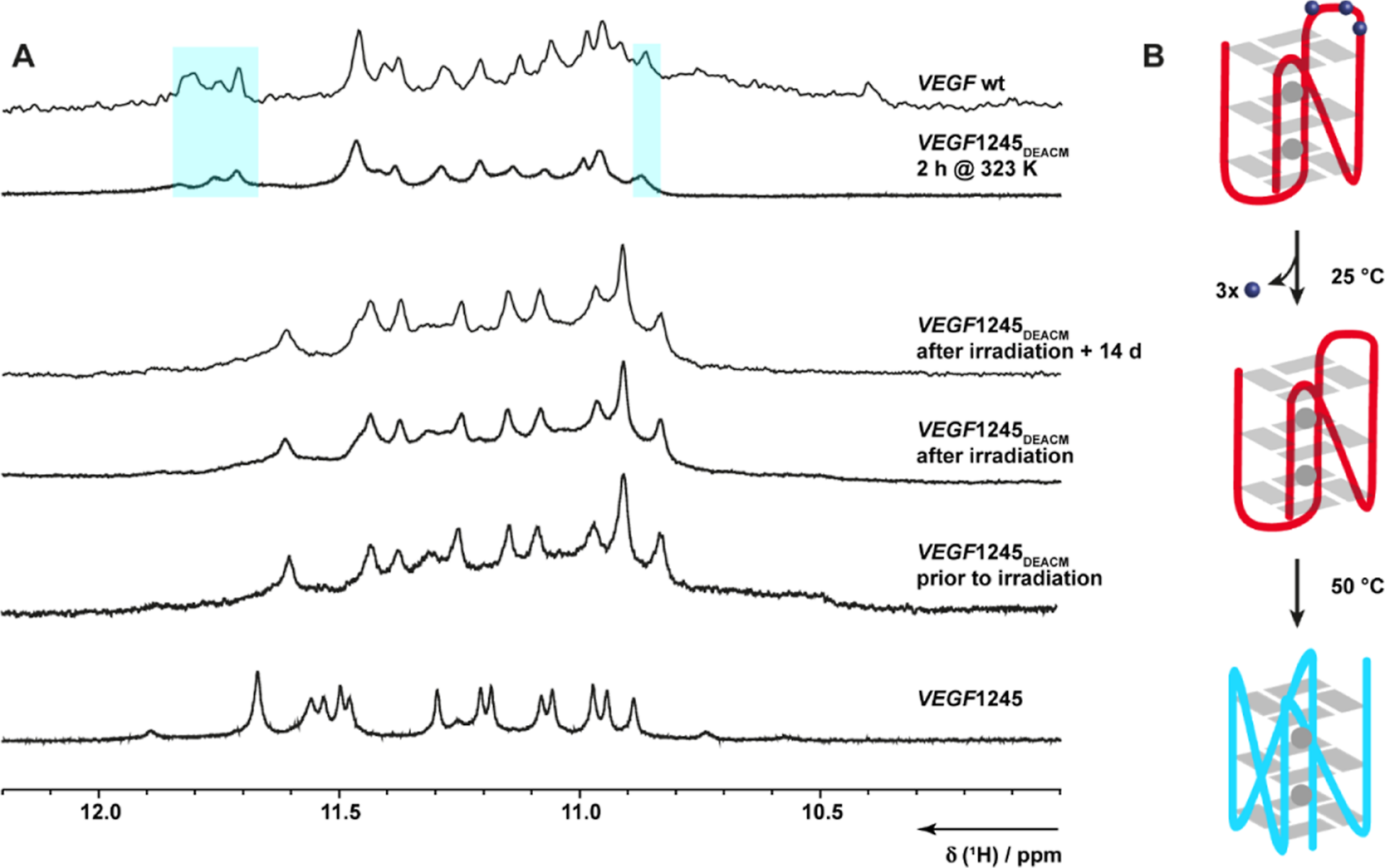
(A) 1D ^1^H NMR spectra of *VEGF*1245 and *VEGF*1245 with photocaged deoxyguanosines within the third G-tract (*VEGF*1245_DEACM_), prior to and after laser irradiation at 355 nm. After application of elevated temperature (50 °C), the thermodynamically stable G4 formed by the *VEGF* wild-type sequence was restored (shown in blue). (B) Schematic representation of the transition of a hybrid G4 fold (red) to the parallel *VEGF*1234 (blue) upon uncaging of the DEACM residues.

Our in vitro characterization reveals that *VEGF*1245 is a metastable G4 structure within the *VEGF* G4 folding landscape. Often, the sequence that is capable of forming a G4 is engaged in duplex DNA and refolding is linked to opening of this duplex DNA, e.g., during replication. While in the case of either spare-tire G4s or G tract capable of register shifts, the existence of one stable structuring is well established, the substantial kinetic inhibition for refolding of a metastable G4 (*VEGF*1245) to the stable G4 (*VEGF*1234) shown here is new. Based on our findings, it is possible that a yet unknown enzymatic mechanism might be required to catalyze such refolding into the thermodynamically most stable conformation. If the transition requires G4 unfolding, a helicase could act as a chaperone for *VEGF*1234. In this case, the helicase has to be specific for the hybrid G4s. To date, most of the reported G4 unfolders are unspecific toward G4 topology. In Saccharomyces cerevisiae, the helicase Pif1 preferentially unfolds antiparallel structures with long loops.^35,36^ Nucleolin, a prominent G4 stabilizing protein, discriminates between parallel G4s with short or long loops.^37^ A specific *VEGF* unwinder is, however, currently not known, and our data suggest follow-up studies to identify such yet unknown protein function.

## Conclusion

In conclusion, we here used NMR and CD spectroscopy to gain insights into the structural aspects of the spare-tire isomer *VEGF*1245, which is formed without the participation of the third G-tract. Such studies are significant because, to date, only the *VEGF*1234 structure has been structurally characterized within the *VEGF* G4 folding landscape. The findings of this study demonstrate that *VEGF*1245 can form a hybrid G4 fold that is achieved only when the third track does no longer contain any deoxyguanosine residue. Notably, this hybrid form does not undergo refolding at temperatures of 25 or 37 °C. This finding indicates that G4 exists in a local energy minimum on the free energy landscape (Figure 6). *VEGF*1245 emerges as a metastable G4 within the *VEGF* G4 folding landscape, raising questions about its functional role in cells. Our findings suggest that an enzymatic process may facilitate its refolding into a more stable conformation, potentially involving a helicase acting as a chaperone for *VEGF*1234. However, such a helicase would need to be specific for hybrid G4s, a property not commonly observed among known G4 unfolding proteins. While certain helicases such as Pif1 and stabilizing proteins such as nucleolin show selective G4 interactions, no specific *VEGF* G4 binding protein has been identified.

**Figure 6:**
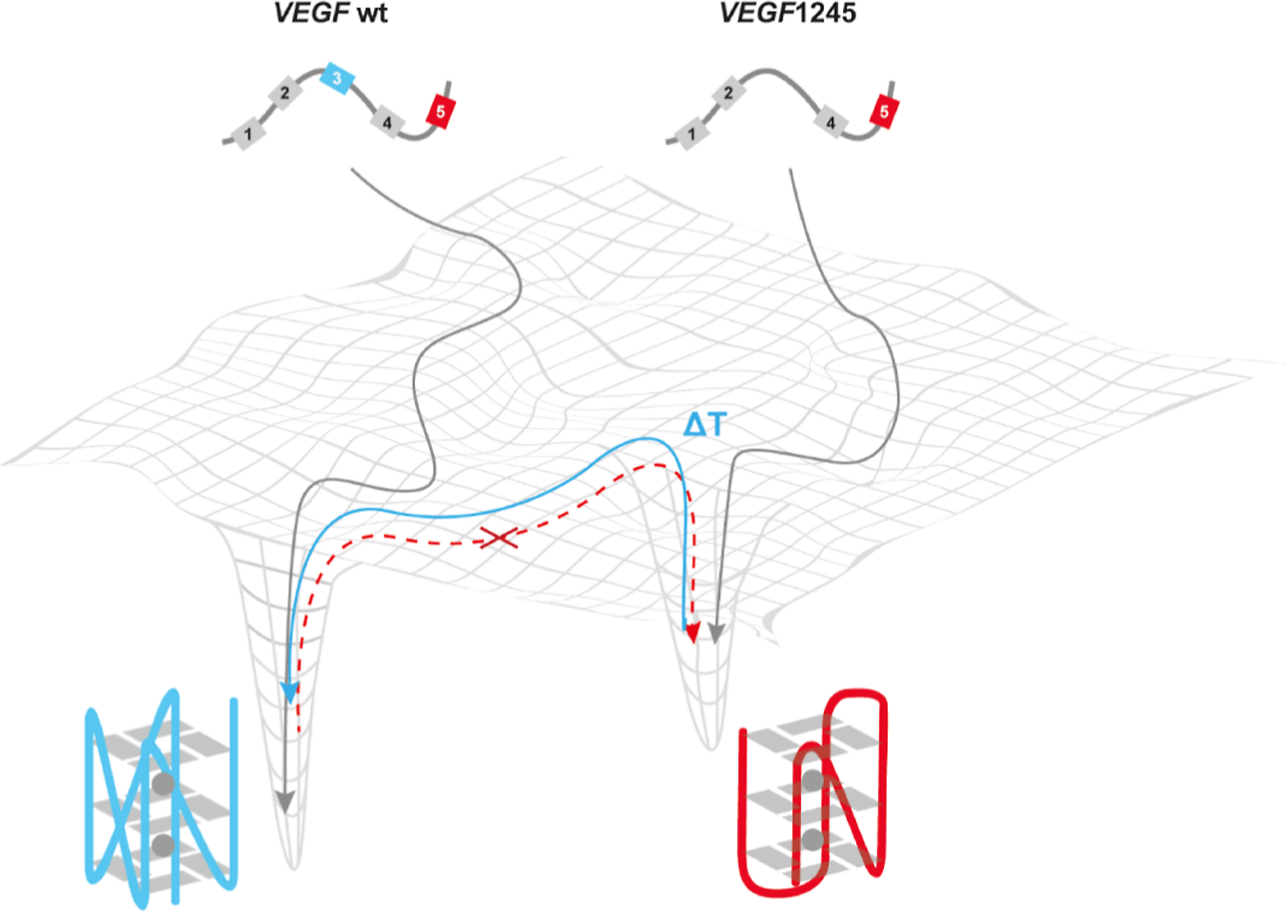
Schematic representation of the free energy landscape of *VEGF* including *VEGF*1245 which can result from oxidation within the third G-tract. The hybrid G4 exists within a local minimum, and with an increase in temperature, the minimum can be overcome and the stable, parallel *VEGF*1234 conformation is reached. This is not possible vice versa.

## Materials and Methods

### Photocaged Oligonucleotide Synthesis

The solid-phase oligonucleotide synthesis of DEACM-caged *VEGF*1245 was performed on an ABI329 DNA/RNA synthesizer from Applied Bioscience on a 1 μmol scale. Commercially available DNA-phosphoramidites, CPG material, and reagents were purchased from LGC Link, empBiotech, Sigma-Aldrich, and Glen Research. The DEACM-caged dG-phosphoramidite was purchased from Glen Research. The phosphoramidites were all 5′-DMTr protected and carried UltraMild protecting groups. They were used at 0.1 M concentrations in dry acetonitrile. A solid support containing a dCAc with 1000 Å pore size was used. 0.3 M BTT in acetonitrile was used as an activator with UltraMild capping reagents (tetrahydrofuran/pyridine/phenoxyacetic anhydride). 5′-Detritylation was performed using 3% TCA in CH_2_Cl_2_. For oxidation, 0.02 M iodine in pyridine/tetrahydrofuran/water (7:2:1) was used. The coupling time for all DNA phosphoramidites was 25 s. The DEACM-caged-dG phosphoramidite was coupled for 6 min. Caged *VEGF*1245 was synthesized with DMTr-OFF. For all works, RNase free water was used. Therefore, 0.1% diethylpyrocarbonate (DEPC) was added to Milli-Q water, stirred overnight, and autoclaved afterward. After synthesis, the solid-support was transferred to a reaction vial, and cleavage and deprotection were carried out using 0.05 M K_2_CO_3_ in methanol for 30 h at RT. Subsequently, desalting was performed using illustra NAP columns (GE Healthcare). The solvent was removed at 4 °C using a vacuum concentrator, and the crude oligonucleotide was then purified by RP-HPLC (details in Supporting Information).

### Folding and Analysis of G4 Structures

Unmodified oligonucleotides were purchased from Eurofins MWG Operon (Ebersberg, Germany) and were of HPLC grade. After desalting via ultracentrifuge filtration devices (Vivaspin, 3 kDa cutoff), the oligomers were dissolved in ddH_2_O and stored at −20 °C. G4 DNA was prepared in 5 or 25 mM potassium phosphate buffer (pH 7.0) and incubated at 95 °C for 5 min. The DNA was folded by slow cooling within 24 h to RT. Selectively ^13^C, ^15^N-labeled oligonucleotides were purchased from INNotope (Innsbruck, Austria) and subsequently HPLC purified with tetrabutylammonium acetate buffer. After this, the oligonucleotides were desalted and prepared as described for unmodified nucleotides.

### NMR Spectroscopy

NMR spectra were recorded on Bruker NMR spectrometers equipped with the probe heads listed in Table 3. All samples were prepared with 100 μM DNA, 0.1 mM 3-(trimethylsilyl)-1-propanesulfonic acid (DSS) as a reference, and 10% D_2_O in 5 or 25 mM potassium phosphate buffer. All NMR experiments were recorded using a jump-return-echo pulse scheme or excitation sculpting to suppress water signals. In situ laser illumination was conducted by a TLL connection to a laser setup (Paladin Advanced 355–8000). Samples were irradiated for 6 s. All NMR spectra were analyzed by Bruker Biospin software TopSpin 4.0.9.

### CD Spectroscopy

Circular dichroism (CD) data were recorded on a Jasco J-810 spectropolarimeter in a 2 mm quartz glass cuvette at 25 °C by using 7.5 μM DNA for each measurement. All curves were smoothed with a Sawitzky–Golay function. Data was collected in a range of 210–320 nm with 5 accumulations. Melting curves were measured between 25 and 95 °C at a rate of 0.5 °C/min at the absorption maximum of the respective sample. Melting curves were fitted by a sigmoidal dose response fit function.

## Supplementary Material



## Data Availability

Experimental raw data are available at https://doi.org/10.25716/gude.1gjr-9wfq.

## Notes

Details on the photocaged DNA synthesis, 2D NMR data for *VEGF*1245, and 1D NMR spectra of the*VEGF* loop and tract variants (PDF)
