# Supplementary material for: Structural Insights into Spare-Tire DNA G‑Quadruplex from the Human VEGF Promoter
Source: ACS Chem Biol. 2025 May 16;20(6):1417–25. doi: 10.1021/acschembio.5c00226 (PMC12186258; doi:10.1021/acschembio.5c00226)
Supplement: Supplementary file 1 [file cb5c00226_si_001.pdf]

Structural Insights into Spare-Tire DNA G-Quadruplex from human VEGF promoter

Ines Burkhart[1], Vivien McKenney[2], Julia Wirmer-Bartoschek[1], J. Tassilo Grün[1], Alexander Heckel[2], Harald Schwalbe[1,*]

1 Institute for Organic Chemistry and Chemical Biology, Center for Biomolecular Magnetic Resonance (BMRZ), Goethe University Frankfurt am Main, 60438 Frankfurt/Main, Hessen, Germany.

2 Institute for Organic Chemistry and Chemical Biology, Goethe University Frankfurt am Main, 60438 Frankfurt/Main, Hessen, Germany.

Supporting Information

1. Details on Photocaged Oligonucleotide Synthesis

1.1 Sequence

Table S1. Sequence of chemically synthesized caged VEGF1245.

	Sequence (5' → 3')
**Caged_VEGF1245**	CGG GGC GGG CCT T**G**^{DEACM}**G**^{DEACM} **G**^{DEACM}CG GGG TCCC GGC GGG GC

1.2 Purification

RP-HPLC

Purification of the crude oligonucleotide was performed on an Agilent 1200 series instrument using a XBridge Peptide BEH C18 OBD Prep Column (300 Å, 5 µm, 10x250 mm) from Waters. Elution was carried out with a solvent-gradient containing solvent A: methanol and solvent B: 400 mM HFIP (hexafluoroisopropanol), 16.3 mM Et3N, pH 7.9, with 4.0 mL/min at 30 °C (Table S2).

Table S2. Gradient used for RP-HPLC purification of caged VEGF1245.

Time / min	%A
**0**	5
**5**	20
**30**	65
**32**	100

1.3 High-resolution mass spectrometry

Purity and identity of caged VEGF1245 was confirmed by analytical RP-HPLC and LC-MS. RP-HPLC was performed on an Agilent 1200 series instrument using a XBridge Peptide BEH C18 column (300 Å, 3.5 µm, 4.6x250 mm) from Waters. Elution was carried out with a solvent-gradient containing solvent A: methanol and solvent B: 400 mM HFIP (hexafluoroisopropanol), 16.3 mM Et3N, pH 7.9, with 0.7 mL/min at 30 °C (Table S2). Mass spectra was obtained on a Thermo Fisher Orbitrap Exploris 120 device.

Table S3. Calculated and found mass for caged VEGF1245.

	Calculated Mass	Found Mass
**Caged VEGF1245**	11015.074	11013.751

A

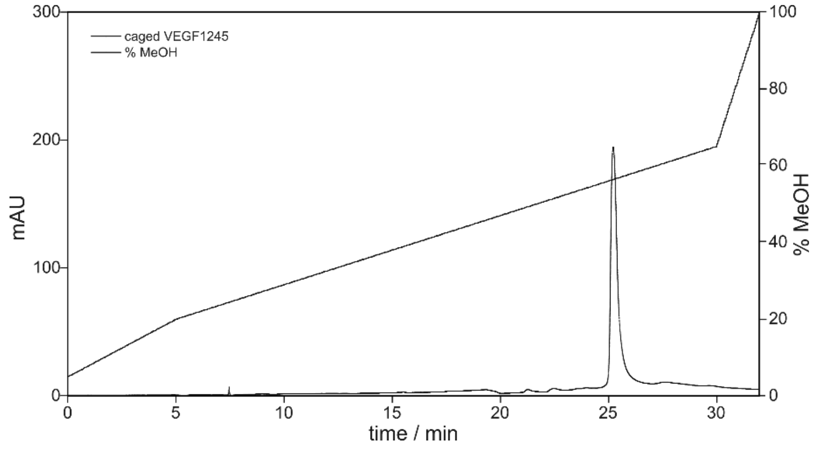

B

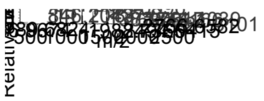

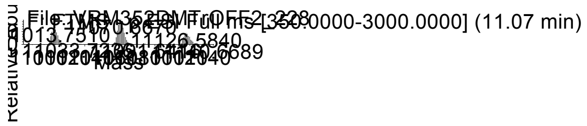

Figure S1. LC-HRMS Data from caged VEGF1245. A) analytical UV RP-HPLC chromatogram with percentage of peak AU B) top: mass spectrum of caged VEGF1245**,** bottom: deconvoluted mass spectrum of caged VEGF1245.

2. Supporting Data

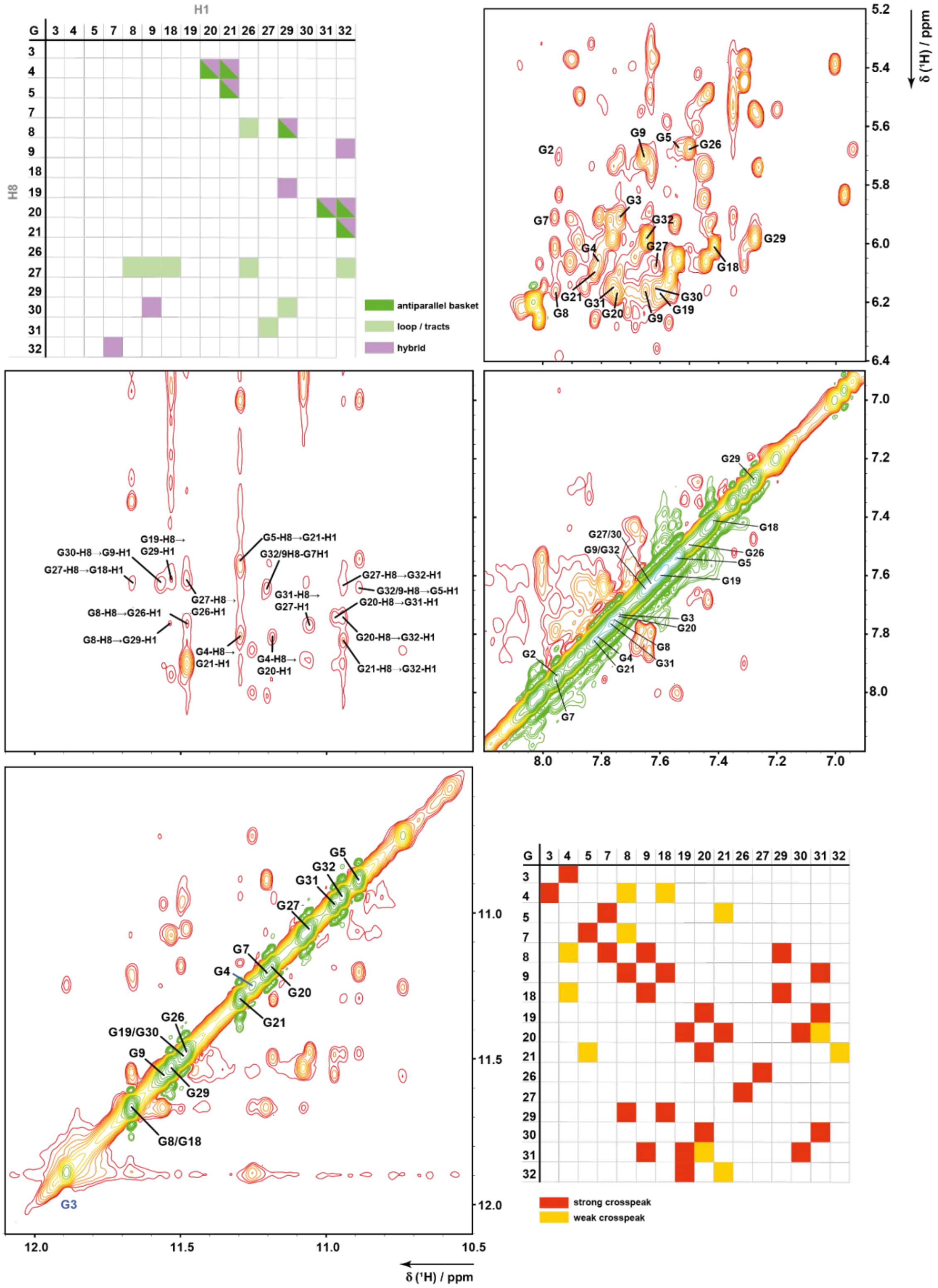

Figure S2. ^{1}H, ^{1}H NOESY experiment of *VEGF*1245, showing imino-imino, imino-aromatic and aromatic-sugar H1 contacts. The table shows strong (red) and weak (yellow) NOE contacts between the imino resonances. The spectrum was recorded with 2048x512 points (700 MHz).

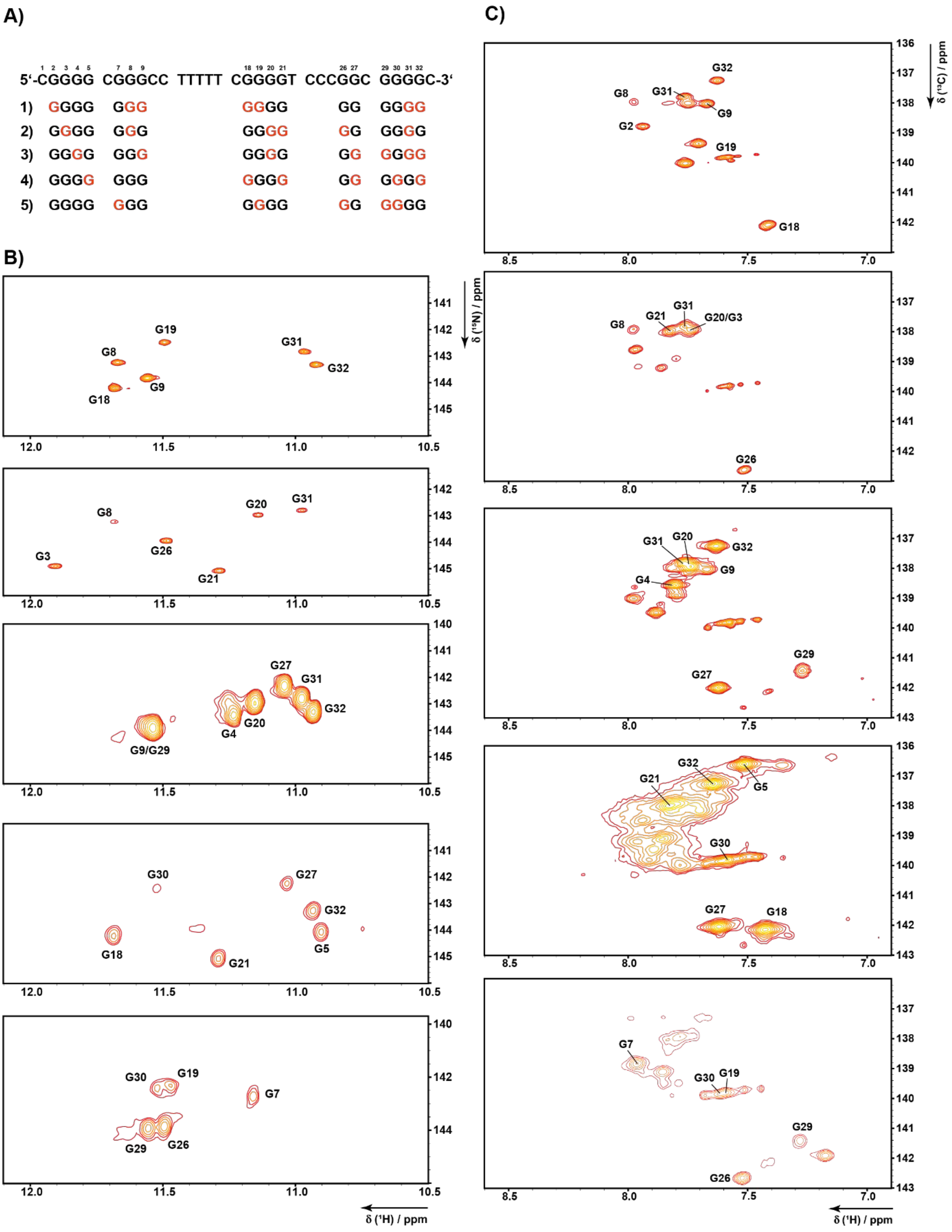
A)
5'-CGGGG CGGGCC TTTTT CGGGGT CCCGGC GGGGC-3'
1) GGGG GGG GGGG GG GGGG
2) GGGG GGG GGGG GG GGGG
3) GGGG GGG GGGG GG GGGG
4) GGGG GGG GGGG GG GGGG
5) GGGG GGG GGGG GG GGGG
B)
G19 G8 G31 G32 G9 G18
G8 G20 G31 G26 G3 G21
G27 G31 G32 G20 G4 G9/G29
G30 G27 G32 G5 G18 G21
G30 G19 G7 G29 G26
δ (¹⁵N) / ppm
δ (¹H) / ppm
C)
G32 G8 G31 G9 G2 G19 G18
G31 G21 G20/G3 G8 G26
G31 G20 G32 G9 G4 G29 G27
G32 G21 G5 G30 G27 G18
G7 G30 G19 G29 G26
δ (¹³C) / ppm
δ (¹H) / ppm

Figure S3. A) Sequences of the used ^{13}C, ^{15}N-labelled samples in this work. Labelled guanosines are marked in red. B) ^{1}H, ^{15}N heteronuclear correlation experiments (BEST-TROSY) and C) ^{1}H, ^{13}C heteronuclear correlation experiments (HSQC) of all sequences with their assignment (800 MHz).

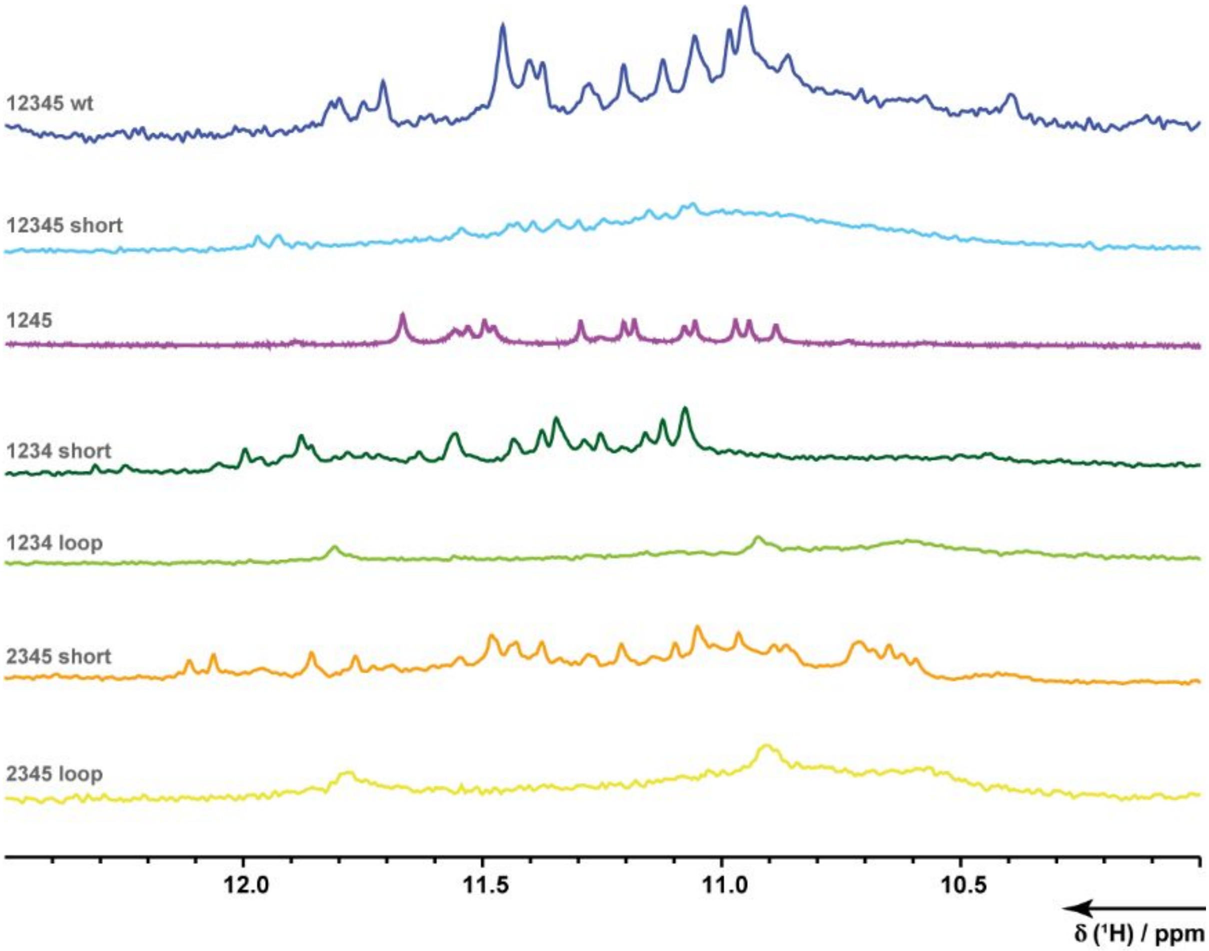

Figure S4. 1D ^{1}H-NMR spectra of VEGF loop and tract variants depicted in Figure 1 and Table 1.
